# Recent Progress in Studies of Porcine Reproductive and Respiratory Syndrome Virus 1 in China

**DOI:** 10.3390/v15071528

**Published:** 2023-07-10

**Authors:** Qi Sun, Hu Xu, Tongqing An, Xuehui Cai, Zhijun Tian, Hongliang Zhang

**Affiliations:** State Key Laboratory for Animal Disease Control and Prevention, Harbin Veterinary Research Institute, Chinese Academy of Agricultural Sciences, No. 678 Haping Road, Xiangfang District, Harbin 150001, China

**Keywords:** PRRSV-1, emergence and prevalence, classification and genomic characteristics, pathogenicity, vaccines and prevention and control

## Abstract

Due to the high incidence of PRRSV mutation and recombination, PRRSV infection is difficult to prevent and control in China and worldwide. Two species of PRRSV, *Betaarterivirus suid 1* (PRRSV-1) and *Betaarterivirus suid 2* (PRRSV-2), exist in China, and PRRSV-1 has always received less attention in China. However, the number of PRRSV-1 strains detected in China has increased recently. To date, PRRSV-1 has spread to more than 23 regions in China. Based on the phylogenetic analysis of ORF5 and the whole genome of PRRSV-1, Chinese PRRSV-1 can be divided into at least seven independent subgroups. Among them, BJEU06-1-like has become the mainstream subgroup in some regions of China. This subgroup of strains has a 5-aa (4 + 1) characteristic discontinuous deletion pattern at aa 357~aa 360 and aa 411 in Nsp2. Previous studies have indicated that the pathogenicity of PRRSV-1 in China is mild, but recent studies found that the pathogenicity of PRRSV-1 was enhanced in China. Therefore, the emergence of PRRSV-1 deserves attention, and the prevention and control of PRRSV-1 infection in China should be strengthened. PRRSV infection is usually prevented and controlled by a combination of virus monitoring, biosafety restrictions, herd management measures and vaccination. However, the use of PRRSV-1 vaccines is currently banned in China. Thus, we should strengthen the monitoring of PRRSV-1 and the biosafety management of pig herds in China. In this review, we summarize the prevalence of PRRSV-1 in China and clarify the genomic characteristics, pathogenicity, vaccine status, and prevention and control management system of PRRSV-1 in China. Consequently, the purpose of this review is to provide a basis for further development of prevention and control measures for PRRSV-1.

## 1. Introduction

**Figure 1:**
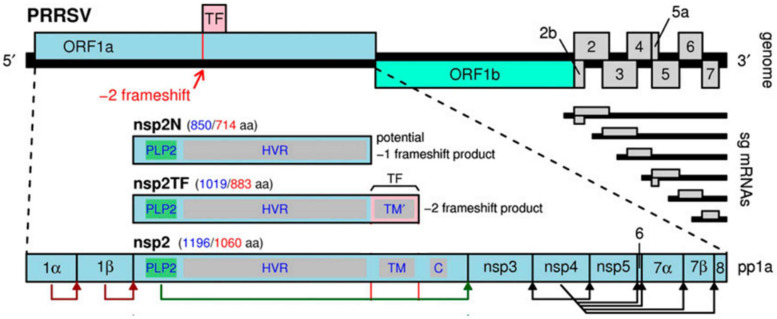
Map of the PRRSV genome [18].

Porcine reproductive and respiratory syndrome (PRRS) is a highly contagious disease causing substantial economic losses in the swine industry worldwide [1], and is caused by porcine reproductive and respiratory syndrome virus (PRRSV). The natural hosts of PRRSV are mainly domestic pigs and wild boars. PRRSV infection causes widespread reproductive failure in pregnant sows and respiratory symptoms in pigs of all ages [2,3,4,5,6]. PRRSV can be transmitted by excreta and secretions of infected pigs [7]. And airborne transmission is also considered to be a potential route of transmission of PRRSV [8,9,10,11]. However, some studies have not reached the same conclusion [12,13]. In addition, the study of Zimmerman et al. showed that PRRSV can also be transmitted through poultry [14]. According to antigenicity, PRRSV is divided into two species, namely, *Betaarterivirus suid 1* (PRRSV-1) and *Betaarterivirus suid 2* (PRRSV-2) [15], which share only 50–70% nucleotide sequence identity [2,16]. The PRRSV genome is approximately 15,000 nucleotides (nt) in length with a 5′-untranslated region (UTR) and a poly(A) tail at the 3′-terminus, which contains at least 11 open reading frames (ORFs), including ORF1a, ORF1b, ORF2a, ORF2b, ORF3-7, ORF5a and ORF2TF (Figure 1) [17,18,19]. Among them, ORF1a and ORF1b encode 16 nonstructural proteins (Nsp) [20]. ORF2TF is a new ORF that was discovered within the Nsp2 region of PRRSV, producing two additional Nsps: Nsp2N and Nsp2TF [18]. The remaining ORF2a, 2b, 3, 4, 5, 5a, 6 and 7 encode eight structural proteins, including GP2, E, GP3, GP4, GP5, ORF5a, M protein and nucleocapsid (N) protein [21,22,23]. Based on the phylogenetic analysis of ORF5 nucleotide sequences and the global PRRSV classification system, PRRSV-1 is divided into three or four subtypes: subtype 1 (subtype Ⅰ (Global)), subtype 2 (subtype Ⅰ (Russia) and subtype II) and subtype 3 (subtype Ⅲ). PRRSV-2 can be divided into nine subtypes: lineage 1 to lineage 9 [24,25,26,27,28]. Although PRRSV-2 is predominant in China, PRRSV-1 isolates have also existed in China for more than twenty years [29]. In recent years, PRRSV-1 has been increasingly isolated in China. To date, PRRSV-1 has spread to more than 23 provinces, municipalities and autonomous regions in China [29,30,31,32,33,34,35,36,37,38,39,40,41,42,43]. To provide a theoretical basis for the prevention and control of PRRS, we summarized and analyzed the genomes of all Chinese PRRSV-1 strains in GenBank, reviewed the prevalence of PRRSV-1 in China, and clarified the genomic characteristics, pathogenicity, vaccine status, and prevention and control management system of PRRSV-1 in China. More detailed information on PRRSV-1 in China is described in this review.

## 2. The Emergence and Prevalence of PRRSV-1 in China

### 2.1. Importation of PRRSV-1 in China

In 1991, the PRRSV-1 strain, Lelystad virus, was first reported and isolated in The Netherlands and is thought to be the prototype strain of PRRSV-1 [44]. Subsequently, Lelystad-like strains were rapidly discovered in France, Belgium, the United Kingdom, Spain and Germany [45]. To date, PRRSV-1 has been mainly prevalent in Europe. However, subtype 1 has spread to countries in other regions, such as Canada [46], the United States [47], China [29], Korea [48] and Thailand [49,50]. The remaining subtypes have been reported only in Eastern European countries [24,27,28].

China mainly imports breeding pigs from the United States, Denmark, France, Britain, Canada and other regions. All these countries have reported the detection of PRRSV-1 [46,47,51,52,53,54]; among these countries, those in breeding pigs imported from Denmark account for the main proportion. Notably, in recent years, a serious outbreak of PRRSV-1 infection has also been reported in Denmark [53]. In addition, the first report on the recombinant strain of two vaccine strains came from France [54] and a PRRSV-1 strain (TZJ2134) with the same recombination pattern had also been reported in China [43]. However, the use of the PRRSV-1 modified live vaccine (MLV) is currently banned in China. Therefore, there is a certain probability that PRRSV-1 could be introduced into China through the import of breeding pigs.

A previous study by Yu et al. suggested that there may have been four events of PRRSV-1 introduction into China from different countries [38]. First, the strains represented by LV-like PRRSV were introduced into China, became widespread and formed independent branches before 2006. Second, strains of PRRSV-1 with high similarity to those in Hungary were introduced into China. Third, it was previously reported that there may have been a small-scale introduction of PRRSV-1 strains similar to the Amervac PRRS vaccine strain into China [34]. The last introduction was prior to 2009, when PRRSV strains similar to those in Denmark were introduced into China, which may have been related to the trade of pigs exported from Denmark to China. The above evidence shows that PRRSV-1 is continuously introduced into and evolving in China.

### 2.2. Emergence and Prevalence of PRRSV-1 in China

**Figure 2:**
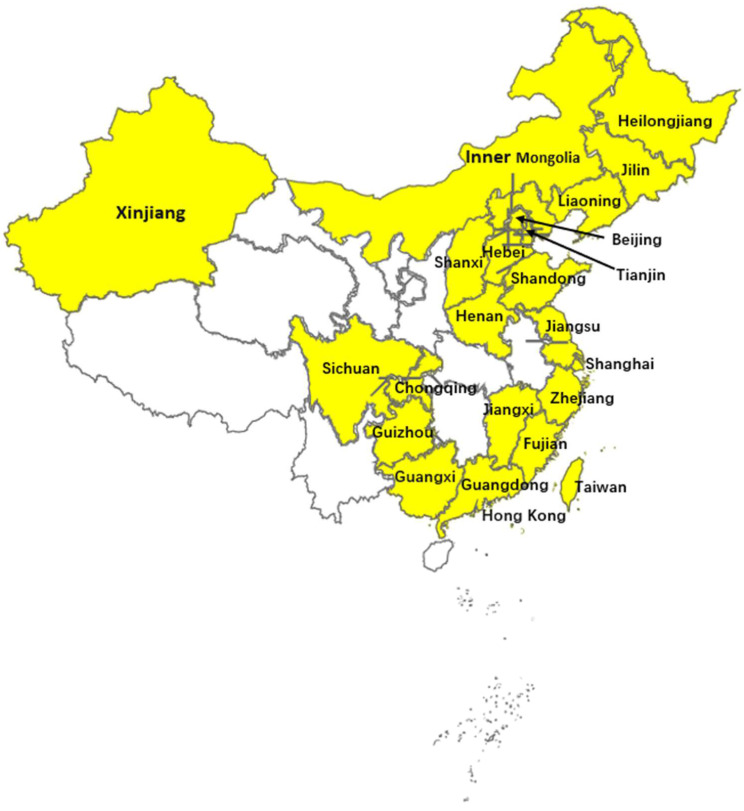
Regions where PRRSV-1 had been detected in China as of March 2023 have been marked with yellow. Map was obtained from https://axhub.im/maps/ (accessed on 19 June 2023).

As early as 1997, Chinese customs intercepted pigs infected with PRRSV-1 (B13, GenBank: AY633973) [55]. This means that the import of PRRSV-1 may have occurred more than 20 years ago. Until 2011, there were only a few Chinese PRRSV-1 partial sequences in GenBank (B13, FJ603). In 2011, Chen et al. isolated PRRSV-1 strains (BJEU06-1, NMEU09-1) from clinical samples collected from 2006 to 2009 in China, which was the first report of wild PRRSV-1 isolates on a pig farm in mainland China [29]. In 2011, Wang X. et al. isolated a PRRSV-1 strain, GZ11-G1, that was highly homologous to the nucleotide sequence of the vaccine strain Amervac [34]. This might have been related to the introduction of vaccinated breeding pigs. Since then, PRRSV-1 infection has been reported continuously in China. Especially in recent years, the number of PRRSV-1 strains detected has increased rapidly in China. In 2016, Zhai et al. collected 750 samples from 50 breeding farms in Guangdong Province, among which the positive rate of PRRSV-1 infection was 24.8% (186/750) [41]. This study has reported the highest rate of positive PRRSV-1 detection in China thus far. In addition, Tan et al. analyzed data on PRRSV-1 detected in primary breeding pig farms and slaughterhouses from the National/OIE PRRS reference laboratory during 2018–2020 and found that the detection rate of PRRSV-1 in primary breeding pig farms was higher than that in slaughterhouses, indicating that the source of PRRSV-1 in China may still be related to introduction, and positive detection in the slaughterhouse continued to be observed, indicating that PRRSV-1 had spread from the original breeding pig farm to the commercial pig farm [56]. Moreover, with the outbreak of African swine fever in recent years in China, the surveillance of animal diseases has been strengthened. This increased surveillance also indirectly leads to an increasing number of reports of PRRSV-1 detection in China. To date, PRRSV-1 infection continues and has been prevalent in at least 23 regions in China. It is widely distributed in central, northern, southern, eastern, northeastern and southwestern China (Figure 2) [29,30,31,32,33,34,35,36,37,38,39,40,41,42,43].

## 3. Classification of PRRSV-1 in China

### 3.1. Phylogenetic Analysis of PRRSV-1 in China

**Figure 3:**
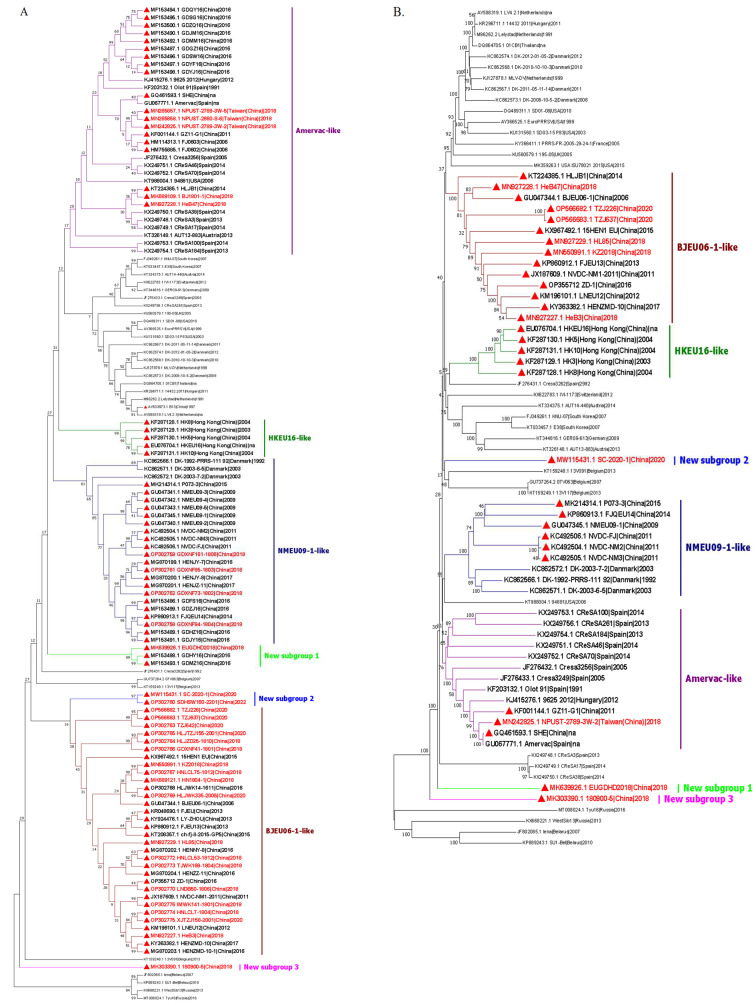
Phylogenetic analysis of the nucleotide sequences of ORF5 and the whole genome of PRRSV-1 strains were performed by using the neighbor-joining method in MEGA 7.0. with 1000 bootstrap replicates for each node. Chinese PRRSV-1 isolates belong to subtype 1 and can be divided into four subgroups (Amervac-like, BJEU06-1-like, HKEU16-like and NMEU09-1-like) and form three new subgroups. (**A**) Phylogenetic analysis of the nucleotide sequences of ORF5 of PRRSV-1 strains. (**B**) Phylogenetic analysis of the nucleotide sequences of the whole genome of PRRSV-1 strains. Chinese PRRSV-1 strains are labeled with red triangles (▲). Chinese PRRSV-1 strains isolated after 2018 are shown in red.

Through the phylogenetic analysis of PRRSV, ORF5 and the whole genome are usually used to classify PRRSV worldwide. PRRSV-1 is commonly divided into three or four subtypes [24,25,26,27,28]. All the strains of PRRSV-1 isolated in China belonged to subtype 1. A previous study showed that Chinese PRRSV-1 could be further divided into four subgroups, including BJEU06-1-like, Amervac-like, HKEU16-like and NMEU09-1-like [39]. Recent studies have shown that BJEU06-1-like has become the main subgroup of PRRSV-1 in some regions of China. In 2022, Li et al. monitored PRRSV in a pig-fattening farm in Henan Province. Of the 38 PRRSV-positive samples, 14 strains were identified as PRRSV-1 (36.94%). All PRRSV-1 strains (14/14) obtained in this pig farm belonged to BJEU06-1-like [33]. Moreover, Xu et al. analyzed the ORF5 gene sequences of 20 strains of PRRSV-1 detected from eight provinces in China during 2016–2022. Among them, 16 PRRSV-1 strains belonged to BJEU06-1-like [37]. To explore the prevalence of each subgroup of Chinese PRRSV-1, all Chinese PRRSV-1 ORF5 sequences and some reference foreign PRRSV-1 ORF5 sequences uploaded on NCBI until March 2023 were analyzed (Figure 3A). The results showed that a total of 38.1% (32/84) of the Chinese PRRSV-1 strains belonged to the BJEU06-1-like subgroup. Among the PRRSV-1 strains detected in the past five years (since 2018) in China, BJEU06-1-like strains accounted for 58.1% (18/31). Moreover, phylogenetic analysis of the whole genome showed that a total of 45.2% (14/31) of the Chinese PRRSV-1 strains belonged to the BJEU06-1-like subgroup and, in the past five years (since 2018) in China, BJEU06-1-like strains accounted for 60% (6/10). This evidence indicated that BJEU06-1-like was the largest subgroup of PRRSV-1 in China and had become the main subgroup of PRRSV-1 in some areas of China. In addition, in the ORF5 phylogenetic analysis, HLJB1 and HeB47 were found to belong to the Amervac-like subgroup. However, both of these strains were found to belong to the BJEU06-1-like subgroup based on the whole genome phylogenetic analysis and the genome was speculated to have undergone recombination events. Intriguingly, according to the phylogenetic analysis of ORF5 sequences, there are three new independent subgroups are formed by Chinese PRRSV-1 strains. Moreover, the phylogenetic analysis of the whole genome showed the same subgroups as the ORF5 analysis (Figure 3B). At present, the strains that have been found to form new independent subgroups have all been detected since 2018, which indicates that Chinese PRRSV-1 has undergone great variation at least during the past five years.

Based on the phylogenetic analysis of both the ORF5 and whole genome sequences of PRRSV-1 strains from China and other countries (Figure 3A,B), the results reveal that the BJEU06-1-like strains and NMEU09-1-like strains in China form two distinct and independent large subgroups, while other subgroup strains are found to be dispersed within larger subgroups that include foreign PRRSV-1 strains. These findings suggest that the BJEU06-1-like and NMEU09-1-like strains have established a stable presence and spread within China. 

### 3.2. The Distribution of Different Subgroups of PRRSV-1 in China

**Figure 4:**
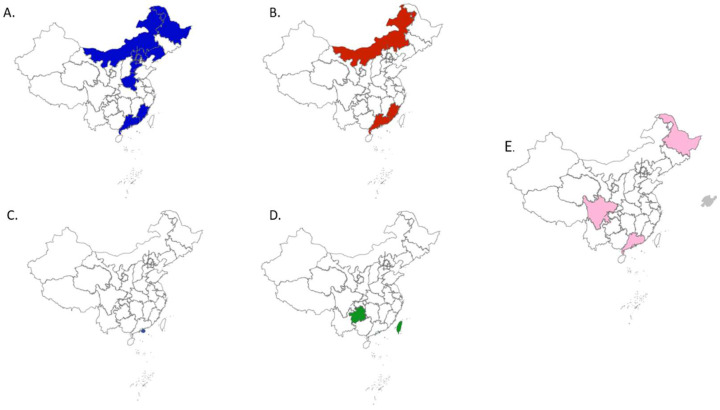
The distribution of each subgroup in China. (**A**) The distribution of BJEU06-1-like strains have been marked with blue. (**B**) The distribution of NMEU09-1-like strains have been marked with red. (**C**) The distribution of HKEU16-like strains has been marked with purple. (**D**) The distribution of Amervac-like strains have been marked with green. (**E**) The distribution of strains forming new subgroups have been marked with pink. Maps were obtained from https://axhub.im/maps/ (accessed on 19 June 2023).

To further clarify the distribution characteristics of each subgroup in China, the regions of the distribution of different subgroups were analyzed using whole genome Chinese PRRSV-1 strains from known collection provinces at NCBI (Figure 4). The results showed that, as the main subgroup of PRRSV-1, the BJEU06-1-like subgroup spread more widely than the other subgroups but most cases were concentrated in the northern regions of China (Figure 4A). This result indicated that BJEU06-1-like strains are mainly prevalent in northern China. We hypothesized that the reason was the poor prevention and control of the BJEU06-1-like strain in northern China or the lack of surveillance data in southern China. Therefore, the prevention, control and monitoring of PRRSV-1 strains should be strengthened in all regions of China. At present, strains of HKEU16-like are only found in Hong Kong and have not yet spread to mainland China (Figure 4C). In contrast, the distribution of strains constituting the new independent subgroups was more dispersed (Figure 4E). At present, Guangdong Province has the most subgroups detected. This indicates that the spread of PRRSV-1 in Guangdong Province is more serious. The above evidence shows that PRRSV-1 was continuously propagated and mutated in China. Therefore, we should strengthen the customs monitoring of pig imports and the monitoring of PRRSV-1 in China to prevent an epidemic of PRRSV-1 in China.

## 4. Genomic Characterization of PRRSV-1 in China

### 4.1. Homology Analysis of PRRSV-1 in China

A previous study reported that the nucleotide substitution rate of PRRSV is the highest among RNA viruses [57]. PRRSV-1 and PRRSV-2 are both pathogens causing PRRS but the homology between them is only 50~70% [2,16]. To explore the genetic and evolutionary trends of PRRSV-1 in China, we analyzed the homology of PRRSV-1 strains with whole genomes published in China (Table 1). The results showed that Nsp2 is the most variable nonstructural protein. In this region, the amino acid homology of PRRSV-1 strains in China was 72.2~99.7%. The residues in Nsp2 have been shown to be associated with many biological characteristics of the virus [32,58,59]. Nsp2 is produced in large quantities in infected cells and collaborates with Nsp3 and Nsp5 to modify the cell membrane of infected cells. In addition, Nsp2 acts as a cofactor for Nsp4 function [60]. Therefore, the variability of NSP2 may affect the corresponding biological function. ORF5a is the most variable structural protein and is a polypeptide comprising 51 amino acids encoded by an alternative ORF of the subgenomic mRNA encoding the major envelope glycoprotein GP5 [21]. The amino acid homology of Chinese PRRSV-1 strains in this region was 72.7~100%. In contrast, the Nsp9 and E proteins are the most conserved nonstructural and structural proteins, respectively. These two regions have less amino acid variation and share higher homology.

To further clarify the trend of the variation in Chinese PRRSV-1 strains among different subgroups and within each subgroup, homology analysis of nucleotide sequences was performed for each subgroup (Table 2). The results showed that there was large variability among the subgroups of PRRSV-1 in China. The genomic homology was 81.5~93.5%. Among them, the BJEU06-1-like strains and NMEU09-1-like strains had large internal variability, and their genomic homology was 86.0~99.7% and 86.1~99.7%, respectively. Moreover, the three Chinese PRRSV-1 strains (EUGDHD2018, SC-2020-1, 180900-5) that constitute the new independent subgroups also showed low homology with other subgroup strains and their genomic homology was 81.5~86.8%. However, the Amervac-like strains and the HKEU16-like strains were less variable, and their genomic homology was 95.1–99.8% and 92.6–97.3%, respectively. Combined with the analysis of the geographical distribution characteristics of each subgroup mentioned above, these results showed that the subgroups with higher variability, such as BJEU06-1-like and NMEU09-1-like, were more widely distributed and dispersed. This indicated that PRRSV-1 strains in China are currently highly variable, and the increase in Chinese PRRSV-1 variability has undoubtedly increased the risk of PRRSV-1 transmission and the difficulty in the prevention and control of PRRSV-1 infection in China.

### 4.2. Recombination of PRRSV-1 in China

Recombination is considered one of the important factors leading to PRRSV genetic diversity. Because PRRSV lacks polymerase proofreading, its replication is prone to errors and mutations. When two different PRRSV strains infect the same cell, parts of their genome sequences can be exchanged during genomic RNA synthesis and then RNA genome recombination can occur [61,62]. RNA recombination has been confirmed to have occurred in cells or pigs coinfected with two different PRRSV strains [63]. In addition, Yu et al. conducted recombination analysis of 91 whole genome sequences of PRRSV-1 from 19 countries during 1991–2018 and found that the high frequency sites of recombination were concentrated in Nsp2 and GP2-GP4 [38]. To further analyze the recombination status of PRRSV-1 strains in China, four PRRSV-1 strains, NMEU09-1, BJEU06-1, Amervac and HKEU16, were used as reference strains, and the whole genome of Chinese PRRSV-1 strains uploaded on NCBI was analyzed by Simplot 3.5.1 and RDP4 (Table 3). The results showed that there were five Chinese PRRSV-1 isolates involved in recombination events (Table 3). The recombinant sites of these Chinese PRRSV-1 recombinant strains were in Nsp2, Nsp3, Nsp9, Nsp10 and GP3-N. Among them, HLJB1 and HeB47 have been reported, showing the recombination of wild strains and vaccine strains [38,39]. The parental strains of HLJB1 include BJEU06-1 and Amervac, and the parental strains of HeB47 include BJEU06-1 and CReSA228. While NVDC-NM2, NVDC-NM3 and NVDC-FJ are recombinations of two wild strains, NMEU09-1 and BJEU06-1, these recombination events have not been reported before. In addition, a recombinant Chinese PRRSV-1 strain TZJ2134 derived from two PRRSV-1 MLV strains (DV strain and Amervac) had been reported in China, but the whole genome was not obtained due to low viral load [43]. Recently, an increasing number of PRRSV-1 recombinant vaccine-related strains have been reported in other countries [51,53,54,64,65]. In 2014, a PRRSV-1 recombinant strain (PRRS-FR-2014-56-11-1) from two PRRSV-1 MLV strains (VP-046BIS and DV strains) was detected on a farm in France (Table 3) [54]. On this farm, two PRRSV-1 MLVs were successively administered a few weeks apart [64]. The recombinant strain showed stronger transmission ability and promoted viremia in pigs more than the two parental strains [64]. In 2019, another PRRSV-1 recombinant strain (DK-2019-10166-107) from two PRRSV-1 MLV strains (Amervac and 96V198) was reported to cause an outbreak in Denmark (Table 3). Similarly, this recombinant strain shows higher transmissibility and pathogenicity than the two parental strains [53]. Due to the prohibition of PRRSV-1 MLV in China, the recombinant strains related to PRRSV-1 vaccine strains that had emerged in China are highly likely to be imported strains.

PRRSV recombinant strains are widely distributed in major pig-producing countries in Asia, North America and Europe. Recombination events within PRRSV-1 or PRRSV-2 are common, but no recombination events between PRRSV-1 and PRRSV-2 have been reported thus far. In addition, some PRRSV-2 strains were reported to be significantly more pathogenic after recombination [66,67]. Moreover, the recombination of PRRSV contributed to its genetic variation and evolution. The continuous recombination of PRRSV may reduce the protective effect of the vaccine and make epidemiological surveillance difficult [68]. Thus, monitoring of the prevalence and pathogenicity of recombinant strains should be strengthened.

### 4.3. Characterization of Nsp2 of PRRSV-1 in China

**Figure 5:**
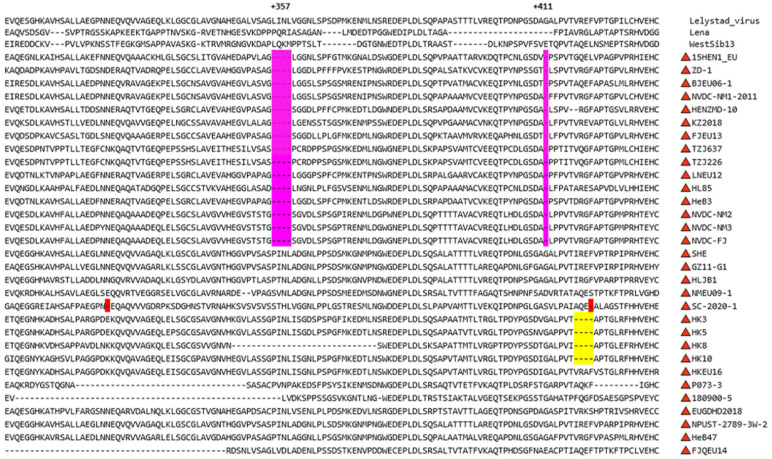
Alignment of the partial Nsp2 protein aa sequences of Chinese PRRSV-1 strains. Purple indicates the characteristic BJEU06-1-like PRRSV-1 5-aa (4  +  1) discontinuous deletion. Yellow indicates the characteristic HKEU16-like PRRSV-1 4 aa continuous deletion (except HKEU16). Red indicates the characteristic SC-2020-1 2-aa (1  +  1) discontinuous deletion. Chinese PRRSV-1 is indicated with a red triangle.

Nsp2 is the most variable protein among the nonstructural proteins of PRRSV. Especially in the central region of Nsp2, PRRSV-1 and PRRSV-2 strains have complex and diverse amino acid (aa) insertion, mutation and deletion patterns acquired during evolution [69]. In 2011, Chen et al. first reported the mutation of Nsp2 and GP3 in Chinese PRRSV-1 [29]. The deletions of these regions are regarded as possible biological markers of the PRRSV-1 variant in China [29]. In 2020, Zhang et al. found 12 different indel patterns when analyzing the Nsp2 region of 19 Chinese PRRSV-1 strains [32]. Among them, the (aa 357~aa 360) + aa411 deletion was the most frequent deletion pattern. In this review, the aa sequences of 34 PRRSV-1 strains (containing 31 Chinese PRRSV-1 strains and 3 foreign representative PRRSV-1 strains that have complete genomes on NCBI) in the Nsp2 region were compared (Figure 5). Interestingly, based on the classification of the ORF5 region, 11 of these 34 PRRSV-1 strains belonged to the BJEU06-1-like subgroup, which all showed a 5-aa (4  +  1) discontinuous deletion pattern at aa 357~aa 360 and aa 411 in the Nsp2 region. In addition, three PRRSV-1 strains (NVDC-NM2, NVDC-NM3 and NVDC-FJ) belonging to NMEU09-1-like also showed the same deletion pattern. These strains were all previously mentioned recombinant strains of the NMEU09-1 strain and BJEU06-1 strain. Moreover, in the HKEU16-like subgroup, except for HKEU16, other strains, such as HK3, HK5, HK8 and HK10, all showed a 4 aa continuous deletion pattern at aa 424~aa 427. Notably, among the Chinese PRRSV-1 strains that formed new subgroups, only SC-2020-1 showed a unique 2-aa (1 + 1) discontinuous deletion pattern at aa 330 and aa 427 in the Nsp2 region. However, other strains showed irregular deletion patterns, which further indicated the high variability of Nsp2.

### 4.4. Characterization of the ORF3/4 Overlapping Region of PRRSV-1 in China

**Figure 6:**
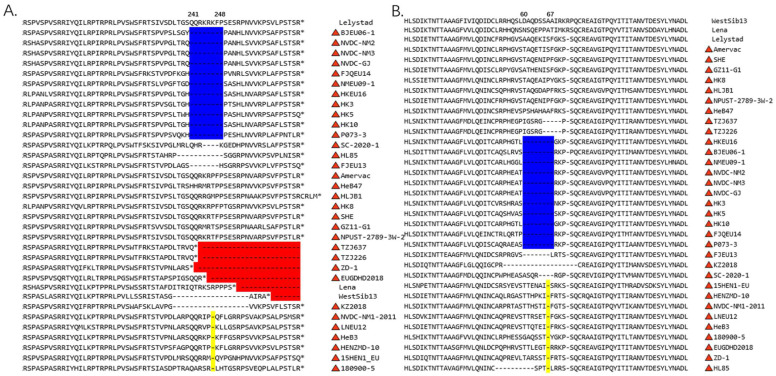
Alignment of GP3 and GP4 protein aa sequences of Chinese PRRSV-1 strains. (**A**) Alignment of the partial ORF3 aa sequences of Chinese PRRSV-1 strains. (**B**) Alignment of the partial ORF4 aa sequences of Chinese PRRSV-1 strains. The aa deletion regions are shown in blue or yellow. Termination codons are indicated by an asterisk (*). The C-terminal truncation mutants are shaded in red. Chinese PRRSV-1 is indicated with a red triangle.

As another hypervariable region in addition to Nsp2, the deletion of the ORF3/4 overlapping region is common in PRRSV-1 isolates [31,32,47,70]. GP3 is a minor structural protein encoded by ORF3 and contains a hypervariable region located at the carboxyl-terminal end (aa 237–252) in the ORF3/4 overlapping region of PRRSV-1 [47]. Previous studies indicated that this region may mutate earlier than other regions under immune selection pressures [71]. Ropp et al. confirmed that there are mutation hotspots at aa 237~aa 252 and aa 57~aa 72 of GP3 and GP4 in the ORF3/4 overlapping region, respectively [47]. Simultaneously, the study of Zhang et al. showed that eight consecutive aa deletions at aa 241~aa 248 were the most common [32]. The deletions of this region usually affect the variation in the length of GP3 and GP4 [32,72]. In this review, the aa sequences of 34 PRRSV-1 strains were compared in the ORF3/4 overlapping region (Figure 6). Notably, the results showed that subtype 1 Chinese PRRSV-1 strain KZ2018 and highly pathogenic subtype 2 strain WestSib13 showed similar deletion patterns in aa 237~aa 254 of GP3 [73]. This indicates that a deletion pattern similar to the highly pathogenic PRRSV-1 strain already exists in China. Moreover, among the Chinese PRRSV-1 strains that formed the new subgroups, only SC-2020-1 showed 4 aa consecutive deletions at aa 244~aa 247 and aa 64~aa 67 of GP3 and GP4.

In addition to deletion patterns, the premature termination of aa of GP3 has been reported abroad before [47]. Amino acid comparison with the Lelystad strain showed that Lena and WestSib13, with high pathogenicity, also have a premature termination at the C-terminus of GP3 [73,74]. Recently, the same premature termination pattern of aa of GP3 was reported in China [33,37]. The studies showed that TZJ226, TZJ637 and ZD-1 had 25-aa and 26-aa premature truncations in the C-terminus of GP3. By analyzing the GP3 of PRRSV-1 strains in China (Figure 6), it was shown that the Chinese PRRSV-1 strain EUGDHD2018 also has a 23 aa C-terminal truncation in GP3. In contrast, HLJB1 has a 4 aa C-terminal late truncation in GP3, which leads to prolonged translation [39]. Previous studies have indicated that the C-terminus of GP3 of PRRSV-1 plays a nonessential role in the life cycle of the virus [47,75,76] and the predicted glycosylation sites are not affected by differences [47]. However, it remains unclear whether premature or late termination at the C-terminus of GP3 will affect the pathogenicity of the virus.

## 5. Pathogenicity of Chinese PRRSV-1

In previous studies, most cases of infections with Chinese PRRSV-1 showed milder clinical symptoms, weaker pathogenicity, lower viral load and fewer clinical symptoms than cases of infections with Chinese PRRSV-2 [34,37,40,77,78,79,80]. As a result, PRRSV-2 has received more attention, while the study of PRRSV-1 has been neglected in China. However, highly pathogenic strains of subtype 1, 2, and 3 PRRSV-1 have been reported [73,74,81,82,83]. In addition, T. Stadejek et al. compared the pathogenicity of Subtype 2 of the PRRSV-1 strains BOR59 and ILI6, and Subtype 1 of the PRRSV-1 strain 18794. The results showed that BOR59 had the strongest pathogenicity and was a highly pathogenic strain, ILI6 had moderate virulence and 18,794 had low virulence. This indicated that the pathogenicity of different strains in the same subtype was also different [84]. The above evidence indicated that some high-pathogenicity PRRSV-1 strains have been reported in other countries [74,81,82,83,84,85,86,87,88]. As the world’s largest pork consumer, China has a considerable demand for breeding pig imports, which indicates that highly pathogenic PRRSV-1 may be at risk of introduction into China with pig trade, similar to how African swine fever was introduced in the past.

Although the number of reports of PRRSV-1 strains isolated in China is gradually increasing, the pathogenic characterization of Chinese PRRSV-1 strains is still limited. To our knowledge, there are only six reports on the pathogenicity of PRRSV-1 in China: HLJB1, GZ11-G1 and NPUST-2789-3W-2 belong to Amervac-like [34,40,78]; ZD-1 and SD1291 belong to BJEU06-1-like [37,79]; and 181187-2 formed a new independent subgroup with PR40/2014 [80]. All six strains showed moderate or low pathogenicity. GZ11-G1 is not lethal to piglets, but it is pathogenic and can cause respiratory symptoms and transient body temperature increases in piglets. It has higher replication efficiency in vivo than the Amervac strain [34]. HLJB1 keeps low viremia and induces a good immune response in infected pigs [78]. NPUST 2789-3W-2 showed low pathogenicity, and analysis of viral loads in the serum of experimental piglets in the high-inoculated (HIN) group and high-inoculated contacted (HC) group showed that Taiwanese wild-type PRRSV-1 had potentially high infectious capabilities [40]. 181187-2 was in the same subtype as the highly pathogenic strain PR40/2014 isolated in Italy, showing moderate pathogenicity. The author speculated that it may have been imported from Italy when the breeding pigs were introduced [80]. SD1291 is an isolate with relatively low pathogenicity, but its replication efficacy in vitro was significantly higher than that of HLJB1 isolated in PAMs [79]. ZD-1 showed moderate pathogenicity to piglets and it has a 26-aa premature truncation in the C-terminus of GP3 [37]. The highly pathogenic PRRSV-1 strain Lena also has a premature termination at the C-terminus of GP3 [74]. However, it is unclear whether these premature terminations in GP3 have any effect on pathogenicity. The pathogenicity data of different PPRRSV-1 strains were compared, as shown in Table 4. In summary, at present, the pathogenicity of PRRSV-1 strains prevalent in China is generally low, but the emergence of 181187-2 and ZD-1 reported in 2023 indicates that the pathogenicity of PRRSV-1 in China has a slight increasing trend. In addition, a high pathogenicity PRRSV-1 strain has been reported both in neighboring countries and in countries important for importing pigs to China [81,86,88]. We should strengthen the monitoring of the PRRSV 1 strain and try to avoid importing pigs from countries with highly pathogenic PRRSV-1 outbreaks.

## 6. Diagnostic Method of PRRSV-1

PRRS detection plays an important role in its clinical prevention and control. In addition to diagnosis through the intuitive clinical symptoms, a range of nucleic acid and antigen/antibody-based assays are currently available for the detection of PRRSV [89] including virus isolation (VI) [90], indirect immunofluorescence assays (IFA) [91], immunoperoxidase monolayer assays (IPMA) [92], serum virus neutralization test (SVN) [93], reverse transcription PCR (RT-PCR) [94,95], quantitative real-time PCR (qPCR) [96], digital PCR (dPCR) [97], recombinase polymerase amplification (RPA) [98], loop-mediated isothermal amplification (LAMP) [99], clustered regularly interspaced short palindromic repeats (CRISPR) [100], metagenomic next-generation sequencing (mNGS) [101] and enzyme-linked immunosorbent assays (ELISA) [102,103]. 

With the rapid development of molecular biology and gene sequencing technology, the RT-PCR detection method has gradually become the mainstream of PRRSV antigen detection methods. At the same time, nested PCR, multiplex PCR, real-time fluorescence quantitative PCR and other detection methods were developed [104,105,106]. At present, there are some reports on the establishment of a variety of RT-PCR detection methods to distinguish PRRSV-2 and PRRSV-1 in China [104,105,106]. These detection methods take a short time and can get the results quickly. It is suitable for the laboratory diagnosis of PRRSV in pigs with acute onset. However, these methods are prone to false detection or missed detection when encountering PRRSV strains with large variability.

Currently, the ELISA is widely used for the detection of PRRSV serological antibodies due to its simplicity, speed, sensitivity and ease of standardization. It is particularly suitable for conducting large-scale epidemiological investigations and monitoring. Numerous commercial ELISA kits are available in the world, with most of them utilizing the PRRSV N protein (Jinnuo and IDEXX) or membrane protein (Hipra) as the coating antigen for indirect ELISA. The IDEXX PRRSV detection method is the most commonly employed worldwide and is generally regarded as the gold standard for detecting PRRSV antibodies [107]. However, these commonly used commercial ELISA kits do not provide specific detection for PRRSV-1 and PRRSV-2. Hipra claims that their two ELISA methods can detect antibodies of both PRRSV-1 and PRRSV-2. However, there have been reports indicating a lack of specificity [108]. On the other hand, Venteo et al. have developed an ELISA method based on the PRRSV-1 nucleocapsid protein which has shown the ability to detect PRRSV-1 infection in the early stages of infection [109]. Currently, the diagnostic methods for PRRSV in China primarily focus on the detection of PRRSV-2 infection, while there are limited methods available for the detection of PRRSV-1 infection. This disparity suggests a need to further develop and improve diagnostic techniques that specifically target PRRSV-1. By expanding the range of available detection methods, the ability to accurately identify and monitor PRRSV-1 infection in China can be improved, so as to better control and prevent PRRSV-1 infection.

## 7. Prevention and Control of PRRSV-1 Infection

### 7.1. Status of the PRRSV-1 Vaccine

Due to the high variability and infectivity of PRRSV, it is widely prevalent worldwide. At present, the main commercialized or developing vaccines used to prevent and control PRRS include inactivated vaccines, MLVs, subunit vaccines, DNA vaccines, and virus vectored vaccines [110]. Among them, the most commonly used PRRSV vaccine in the world is the PRRSV MLV, which was the first commercial PRRS vaccine launched in the United States in 1994 [111]. In the late 1990s, PRRSV-1 MLV was used to prevent PRRSV-1 infection in Europe [112]. To date, it is also allowed in some Asian countries, such as Vietnam, Korea, and Taiwan in China. Common PRRSV-1 MLVs worldwide include Porcilis PRRS (MSD Animal Health), Amervac PRRS (Hipra), ReproCyc PRRS EU (Boehringer Ingelheim), Pyrsvac-183 (SYVA Laboratories), Ingelvac PRRSFLEX^®^ EU (Boehringer Ingelheim), Suvaxyn^®^ PRRS MLV (Zoetis), and Unistrain^®^ PRRS (Hipra) (Table 5) [110,113]. PRRSV-MLVs can reduce the spread of viruses in infected pigs and induce a late but effective protective immune response to homologous wild-type strains. However, PRRSV-MLVs confer only partial protection or no protection against heterologous strains [114,115]. In addition, PRRSV-MLVs have the risk of producing vaccine strains or recombining with wild strains during replication. As a result, the safety of these vaccines is challenged [116,117]. The emergence of vaccine-associated strains undoubtedly increased the difficulty in the prevention and control of PRRSV-1 infection in China. Therefore, these results remind us that it is very important to rationally use the vaccine, and further strengthen the domestic management of PRRSV-1 MLV and the monitoring of imported animal diseases.

### 7.2. Prevention and Control of PRRSV-1 Infection in China

With the increasing genetic and variation diversity of PRRSV-1 in China, new strains appear frequently, and the subgroups of existing strains are complex, which makes the prevention and control of PRRSV-1 more difficult in China. Due to the low pathogenicity of PRRSV-1 prevalent in China, it has received less attention. There is also a lack of effective detection methods for PRRSV-1 in clinical practice, which may lead to the missed detection of PRRSV-1. At present, the focus of the prevention and control of PRRSV infection in China is to prevent and control PRRSV-2 infection through vaccination. However, the commercial diagnostic reagents and vaccine reserves for PRRSV-1 are insufficient, and strategies such as biosafety management are mainly adopted to prevent and control PRRSV-1 infection in China. A good feeding mechanism should be established, balanced nutrition should be maintained, and a suitable feeding environment for the pig farm and a reasonable feeding density should be ensured. It is necessary to strictly control the flow of pigs and ensure the standardization of pig transportation operations. The source of infection should be isolated, biosafety prevention and control should be performed effectively, the scientific use of vaccines should be standardized, reasonable virus treatment programs should be formulated, and the prevalence of PRRSV-1 in various regions should be regularly monitored. More research is needed to better understand the biological characteristics of PRRSV-1 in China to achieve effective prevention and control of PRRSV infection.

## 8. Conclusions

To date, the published literature has indicated that PRRSV-1 has been detected in at least 23 regions in China. Among them, the BJEU06-1-like subgroup has become the main subgroup of PRRSV-1 in some regions of China, showing a characteristic 5-aa (4 + 1) discontinuous deletion pattern at aa 357~aa 360 and aa 411 in NSP2. In addition, PRRSV-1 strains in China currently have great variability. The high incidence of PRRSV mutation and recombination, and a lack of effective detection methods for PRRSV-1 in China undoubtedly increased the difficulty in the prevention and control of PRRSV-1 infection in China. PRRSV-1 strains had low to moderate pathogenicity. However, the use of the PRRSV MLV is currently banned in China. Therefore, there is an urgent need to monitor PRRSV-1 in China. We should focus on improving and strengthening the establishment of animal feeding management and biosafety systems, strengthen quarantine and monitoring of epidemic disease, and adopt comprehensive prevention and control measures combining the use of vaccines and provenance purification strategically.

## Figures and Tables

**Table 1:** Amino acid identity of nonstructural and structural proteins of Chinese PRRSV-1 strains (%).

ORF	Cleavage Products	BJEU06-1	NMEU09-1	Amervac	HKEU16	Chinese PRRSV-1 Strains
ORF1a	Nsp1α	86.1~95.6%	89.4~96.1%	90.0~100%	87.2~100%	85.6~100%
	Nsp1β	76.6~100%	74.6~82.9%	75.6~100%	71.7~99.5%	71.2~100%
	Nsp2	76.6~93.0%	72.4~83.7%	77.0~99.7%	74.0~96.0%	72.2~99.7%
	Nsp3	91.3~96.1%	91.7~95.7%	91.7~100%	92.2~100%	90.4~100%
	Nsp4	86.2~95.1%	83.3~93.1%	88.2~100%	87.2~99.0%	83.3~100%
	Nsp5	88.2~98.2%	84.7~94.1%	88.8~100%	87.1~97.6%	84.7~100%
	Nsp6	93.8~100%	93.8~100%	93.8~100%	87.5~100%	87.5~100%
	Nsp7α	92.6~98.7%	92.6~98.7%	91.9~100%	91.3~100%	91.3~100%
	Nsp7β	86.7~99.2%	85.0~96.7%	90.8~100%	88.3~98.3%	84.2~100%
	Nsp8	91.1~100%	86.7~100%	88.9~100%	82.2~100%	82.2~100%
ORF1b	Nsp9	94.7~99.8%	94.9~96.9%	95.7~100%	95.7~98.9%	93.8~100%
	Nsp10	93.4~98.2%	91.6~96.6%	93.0~100%	90.5~96.6%	90.5~100%
	Nsp11	96.4~99.1%	93.3~97.8%	94.6~100%	94.2~100%	93.3~100%
	Nsp12	89.5~97.4%	86.3~95.4%	90.2~100%	89.5~99.3%	86.3~100%
ORF2a	GP2	87.6~99.2%	84.8~92.8%	86.4~100%	86.0~98.0%	84.4~100%
ORF2b	E	93.0~100%	91.5~100%	91.5~100%	87.3~98.6%	87.3~100%
ORF3	GP3	82.8~93.7%	82.4~91.2%	84.6~100%	82.8~95.0%	80.8~100%
ORF4	GP4	86.4~94.1%	85.8~96.6%	82.5~98.9%	83.5~96.6%	80.3~100%
ORF5	GP5	83.7~94.1%	83.7~92.6%	85.6~98.0%	84.2~99.0%	80.7~100%
ORF5a	ORF5a	79.5~93.2%	79.5~100%	79.5~100%	77.3~95.5%	72.7~100%
ORF6	M	89.1~95.4%	89.7~95.4%	90.8~99.4%	89.1~100%	87.4~100%
ORF7	N	82.9~94.6%	86.0~94.6%	87.6~100%	85.3~100%	82.9~100%

The amino acids in Nsp3, Nsp8 and Nsp9 of EUGDHD2018 were not corrected, and EUGDHD2018 was removed when amino acid alignment was performed in these regions.

**Table 2:** Nucleotide sequence identity of different subgroups and within each subgroup of Chinese PRRSV-1 strains (%).

Subgroups	BJEU06-1-Like	NMEU09-1-Like	Amervac-Like	HKEU16-Like	180900-5	SC-2020-1	EUGDHD2018
BJEU06-1-like	86.0~99.7%						
NMEU09-1-like	82.2~87.6%	86.1~99.7%					
Amervac-like	85.5~93.5%	84.8~87.1%	95.1~99.8%				
HKEU16-like	84.8~89.1%	83.6~85.1%	87.9~89.4%	92.6~97.3%			
180900-5	81.7~84.2%	81.5~82.6%	83.7~84.5%	83.2~83.6%			
SC-2020-1	83.1~86.0%	82.5~83.3%	85.2~86.4%	84.9~85.1%	82.0%		
EUGDHD2018	82.7~85.5%	82.1~82.8%	85.5~86.8%	84.1~84.3%	81.7%	82.2%	

**Table 3:** Chinese PRRSV-1 recombinant strains and foreign PRRSV-1 recombinant vaccine strains.

Strains	Year	Country	Breakpoints		Parental Sequence		Reference
			Beginning	Ending	Minor	Major	
NVDC-NM2	2011	China	917	3416	BJEU06-1	NMEU09-1	None
NVDC-NM3	2011	China	917	3416	BJEU06-1	NMEU09-1	None
NVDC-FJ	2011	China	917	3416	BJEU06-1	NMEU09-1	None
PRRS-FR-2014-56-11-1	2014	France	4972	8430	DV	VP-046BIS	[54]
			500	1370			
			3646	4272			
			1834	1952			
HLJB1	2017	China	843	2132	BJEU06-1	Amervac	[39]
HeB47	2018	China	12,500	14,000	CReSA228	BJEU06-1	[38]
DK-2019-10166-107	2019	Denmark	12,604	15,062	Amervac	96V198	[53]
TZJ2134	2021	China	9397	11,266	DV	Amervac	[43]

**Table 4:** Comparison of the pathogenicity of different PRRSV-1 strains.

Infected PRRSV Strain	Subtype	Country	Days of Fever	Clinical Symptoms	Pathological and Histopathological Lesions	Viremia	Reference
NPUST-2789-3 W-2	Subtype 1	China	No fever	Mild clinical symptoms	Obvious pathological changes	Peaked at 14 dpi	[40]
ZD-1	Subtype 1	China	6 days (≥40 °C)	Medium clinical symptoms	Obvious pathological changes	Peaked at 7 dpi, longer than 21 days	[37]
HLJB1	Subtype 1	China	3 days (≥40 °C)	Mild clinical symptoms	Obvious pathological changes	Peaked at 7 dpi, longer than 21 days	[78]
GZ11-G1	Subtype 1	China	3 days (≥40 °C)	Mild clinical symptoms	Mild pathological changes	Peaked at 7 dpi, longer than 21 days	[34]
PR40/2014	Subtype 1	Italy	19 days (≥40 °C)	Severe clinical symptoms, 3/7 pigs died	Severe pathological changes	Peaked at 7 dpi, longer than 35 days	[83]
WestSib13	Subtype 2	Russia	No fever	Severe clinical symptoms, 5/5 pigs died	Severe pathological changes	Peaked at 6 dpi, all pigs died seven days after infection	[73]
Lena	Subtype 3	Belarusian	25 days (≥40 °C)	Severe clinical symptoms, 4/10 pigs died	Severe pathological changes	Peaked at 14 dpi, longer than 35 days	[74]

**Table 5:** Common commercial PRRSV-1 MLV vaccines.

Vaccine	Parental Strain	Species	Producer/Developer
Ingelvac PRRSFLEX^®^ EU	94881	PRRSV-1	Boehringer Ingelheim
ReproCyc^®^ PRRS EU	94881	PRRSV-1	Boehringer Ingelheim
Pyrsvac-183^®^	All-183	PRRSV-1	Syva
Unistrain^®^ PRRS	VP-046 BIS	PRRSV-1	Hipra
Amervac^®^ PRRS	VP-046	PRRSV-1	Hipra
Porcilis^®^ PRRS	DV	PRRSV-1	MSD Animal Health
Suvaxyn^®^ PRRS MLV	96V198	PRRSV-1	Zoetis

## Data Availability

Not applicable.
